# Varenicline Prevents LPS-Induced Inflammatory Response via Nicotinic Acetylcholine Receptors in RAW 264.7 Macrophages

**DOI:** 10.3389/fmolb.2021.721533

**Published:** 2021-10-12

**Authors:** Elif Baris, Hande Efe, Mukaddes Gumustekin, Mualla Aylin Arici, Metiner Tosun

**Affiliations:** ^1^ Department of Pharmacology, Graduate School of Health Sciences, Dokuz Eylul University, Izmir, Turkey; ^2^ Department of Pharmacology, Faculty of Medicine, Izmir University of Economics, Izmir, Turkey; ^3^ Department of Medical Biology and Genetics, Graduate School of Health Sciences, Dokuz Eylul University, Izmir, Turkey; ^4^ Department of Medical Pharmacology, Faculty of Medicine, Dokuz Eylul University, Izmir, Turkey

**Keywords:** varenicline, α7nAChR, inflammation, cytokine, proliferation, migration

## Abstract

The cholinergic anti-inflammatory pathway plays an important role in controlling inflammation. This study investigated the effects of varenicline, an α7 nicotinic acetylcholine receptor (α7nAChR) agonist, on inflammatory cytokine levels, cell proliferation, and migration rates in a lipopolysaccharide (LPS)-induced inflammation model in RAW 264.7 murine macrophage cell lines. The cells were treated with increasing concentrations of varenicline, followed by LPS incubation for 24 h. Prior to receptor-mediated events, anti-inflammatory effects of varenicline on different cytokines and chemokines were investigated using a cytokine array. Nicotinic AChR–mediated effects of varenicline were investigated by using a non-selective nAChR antagonist mecamylamine hydrochloride and a selective α7nAChR antagonist methyllycaconitine citrate. TNFα, IL-1β, and IL-6 levels were determined by the ELISA test in cell media 24 h after LPS administration and compared with those of dexamethasone. The rates of cellular proliferation and migration were monitored for 24 h after drug treatment using a real-time cell analysis system. Varenicline decreased LPS-induced cytokines and chemokines including TNFα, IL-6, and IL-1β via α7nAChRs to a similar level that observed with dexamethasone. Varenicline treatment decreased LPS-induced cell proliferation, without any nAChR involvement. On the other hand, the LPS-induced cell migration rate decreased with varenicline via α7nAChR. Our data suggest that varenicline inhibits LPS-induced inflammatory response by activating α7nAChRs within the cholinergic anti-inflammatory pathway, reducing the cytokine levels and cell migration.

## Introduction

Lipopolysaccharide (LPS), an endotoxin of Gram-negative bacteria, induces “pathogen-associated molecular pattern (PAMP) recognition receptors” (toll-like receptors, TLRs expressed on immune system cells. Exposure to LPS initiates an inflammatory response that includes unrestrained production of pro-inflammatory cytokines: tumor necrosis factor (TNF), interleukins (IL-1, IL-6, and IL-8), and platelet-activating factor (PAF) from immune cells (Palsson-McDermott and O’Neill, 2004; Murdock and Núñez, 2016).

Uncontrolled pro-inflammatory cytokine release initiates systemic inflammatory response that can be regulated by the cholinergic system, which is crucial for balancing the body’s response to inflammation and survival. The control of inflammation through the parasympathetic system is called the cholinergic anti-inflammatory pathway (CAP). The CAP is defined as a comprehensive neural mechanism that attenuates pro-inflammatory cytokine release through the vagus nerve and activation of nicotinic receptors expressed on mononuclear phagocytic cells such as lymphocytes, macrophages, mast cells, dendritic cells, basophils, and microglia (Fujii et al., 2017; Snider et al., 2018). The pharmacological activation of cholinergic receptors or electrical stimulation of the vagus nerve limits pro-inflammatory cytokine production in various models (i.e., ischemia, pancreatitis, colitis, hemorrhage, and sepsis). Clinical studies showed that agents acting on the cholinergic anti-inflammatory pathway improved sequential organ failure and mortality rates in patients with sepsis (Zimmermann et al., 2017; Pinder et al., 2019a; Pinder et al., 2019b). Most of the studies have focused on pharmacological interventions to activate cholinergic receptors, particularly α7nAChR, and their expression on immune cells is required for communication between the cholinergic system and the immune system cells. The inhibitory effect of α7nAChR agonists on TNFα, IL-1, IL-6, and IL-8 plays an important anti-inflammatory role. Studies showed that α7nAChR agonists, such as nicotine, acetylcholine, choline, or GTS-21 modulate LPS-induced pro-inflammatory cytokines, promote and improve survival in different experimental models (Borovikova et al., 2000; Pavlov et al., 2007; Fujii et al., 2017; Zimmermann et al., 2017; Snider et al., 2018). Thus, α7nAChR-activating agents appear to have therapeutic efficacy in inflammatory processes (Borovikova et al., 2000; Bernik et al., 2002; De Jonge and Ulloa, 2007; Zimmermann et al., 2017; Pinder et al., 2019a; Pinder et al., 2019b).

Following LPS-induced TLR activation, macrophages migrate into inflamed tissues and eliminate pathogens via phagocytosis. LPS-activated macrophages produce different cytokines and chemokines including macrophage inflammatory proteins, monocyte chemoattractant proteins, TNFα, IL-1β, and IL-6, which in turn stimulate macrophage migration (Toh et al., 2006; Yang et al., 2015). On the other hand, acetylcholine reportedly inhibits LPS-induced matrix metalloproteinase 9 (MMP-9) production and macrophage migration via α7nAChR activation (Yang et al., 2015).

Widely used as an efficient and safe therapeutic option for smoking cessation, varenicline reportedly has potent and full agonistic properties on α7nAChRs, besides having partial agonistic effects on α4β2-nAChRs (Mihalak et al., 2006a; 2006b; Hays et al., 2008). A recent clinical study has demonstrated that 12-week varenicline treatment modulates inflammation and oxidative damage (McElroy et al., 2018). Furthermore, immunohistochemical experiments have shown that varenicline treatment suppressed inflammation and the number of immune system cells, via α7nAChR activation in the brain and lung tissues in an animal model of ischemia and emphysema (Chen et al., 2017; Koga et al., 2018). However, there is no clear explanation on the role of varenicline in LPS-induced inflammatory response and macrophage migration. Therefore, this study investigated the effects of varenicline on α7nAChR–mediated activation of CAP, cell proliferation, and migration induced by LPS.

## Material and Methods

### Cell Culture

RAW 264.7 murine macrophage cells (ATCC TIB-71, Manassas, VA, United States) were maintained in DMEM (Sigma Aldrich D6429), supplemented with heat-inactivated FBS (10%) and penicillin (100 U/ml) and streptomycin (100 μg/ml, Gibco, Carlsbad, CA) at 37°C in a 5% CO_2_ incubator. Regular checks for *Mycoplasma* contamination were performed with a mycoplasma detection kit (Biowest, Riverside, MO, United States). The cells (500,000/well) were seeded in 48-well tissue culture plates after detachment with scraping incubated for 24 h in serum-free media for reattachment to the surface. Before adding chemicals, the medium was replaced with fresh serum-free media. In the first group, the cells were treated with LPS (*Escherichia coli*, Sigma-Aldrich L4130 0111: B4) at various concentrations (in μg/ml: 0.5–2.5–4 and 5) to determine the effective concentration at which cytokines are released (Parrish et al., 2008). In the second group, the cells were pretreated with varenicline tartrate (Sigma-Aldrich PZ0004) with increasing concentrations (in μM: 1-3-10-30) 30 min prior to LPS administration to determine effective varenicline concentration on LPS-induced cytokine levels. Additionally, the anti-inflammatory effect of varenicline was compared with that of dexamethasone (0.1 μΜ, Sigma-Aldrich D4902) (Ai et al., 2020). In the third group, to investigate the involvement of nicotinic receptors, a non-selective nicotinic ACh receptor antagonist mecamylamine hydrochloride (MEC, 50 μΜ, Sigma-Aldrich M9020) and a selective α7nAChR antagonist methyllycaconitine citrate (MLA, 1 μΜ, Sigma-Aldrich M168) were applied 30 min before varenicline and LPS administration (Yang et al., 2015; Yi et al., 2015). RAW 264.7 cells at passage #5 were originally obtained from ATTC (gift).

### Protein Analyses

The levels of TNFα, IL-1β, and IL-6 released into the culture media 24 h after LPS administration were determined by the enzyme-linked immunosorbent assay (ELISA) (Invitrogen, Carlsbad, CA) and cytokine array (Proteome Profiler^TM^ Array, Panel A, R, and D System, Minneapolis, MN, United States) according to the manufacturer’s instructions.

### Proliferation and Migration Assays

A real-time cell analysis system (xCELLigence RTCA DP “dual purpose,” Acea Biosciences, San Diego) was used to monitor proliferation or migration in real time in different plates and experimental settings. E-plate 16, used for proliferation assay, is a single-use cell culture plate with highly sensitive gold microelectrodes. Real-time changes in electrical impedance were expressed as cell index (CI).The background impedance of 100 µL medium was measured prior to seeding cells (10,000 cells/well). The cells were incubated at room temperature for 30 min before running RTCA. After 24-h proliferation, cells were treated with LPS (4 μg/ml) with or without varenicline (1 µM). The change in cell proliferation was monitored for 48 h at 15-min intervals. Negative control groups (cell-free culture medium) were tested in each plate.

The migration assay was also performed using the xCELLigence RTCA DP instrument, which allows us to monitor cell invasion and migration (CIM) through the inner microporous membrane assembled within an integrated Boyden chamber. As in the proliferation assay, real-time changes in electrical impedance were also recorded. Before the experiments, the cells were washed with a serum-free medium 24 h before the experiment and then seeded (30,000 cells/well) in the upper migration chamber (CIM plate) with the serum-free medium and incubated at room temperature for 30 min. The lower chambers were filled with a serum-containing medium, and the upper chambers with serum-free medium. The number of cells migrated from the upper to the lower chamber was determined real time for 24 h with 15-min intervals after drug treatment. The change in the cell migration rate was expressed as cell index (CI) (Bird and Kirstein, 2009; Cano et al., 2016; Selli et al., 2016).

In the first migration assay group, the cells were treated with LPS (4 μg/ml) in the presence or absence of varenicline (1 µM). The second group was treated with a non-selective nAChR antagonist MEC (50 μΜ) and/or a selective α7nAChR antagonist MLA (1 μΜ), both of which were applied 30 min before varenicline and LPS administration. The change in the cell migration rate was monitored for 48 h at 15-min intervals. Negative control groups (serum- and cell-free culture medium) were tested in each plate, as reported previously (Bird and Kirstein, 2009; Cano et al., 2016).

### Statistical Analysis

One-way analysis of variance analysis (ANOVA) with post hoc Tukey–Kramer multiple comparison tests or Student’s *t*-test (GraphPad Prism 5, La Jolla, CA) were used to compare means. Data were expressed as mean *±* S.E.M (*n* = 5–7, each performed in triplicate), and a *p* value *<* 0.05 was accepted as statistically significant.

## Results

### Inhibitory Effects of Varenicline on LPS-Induced Cytokine Secretions From Macrophages

**FIGURE 1:**
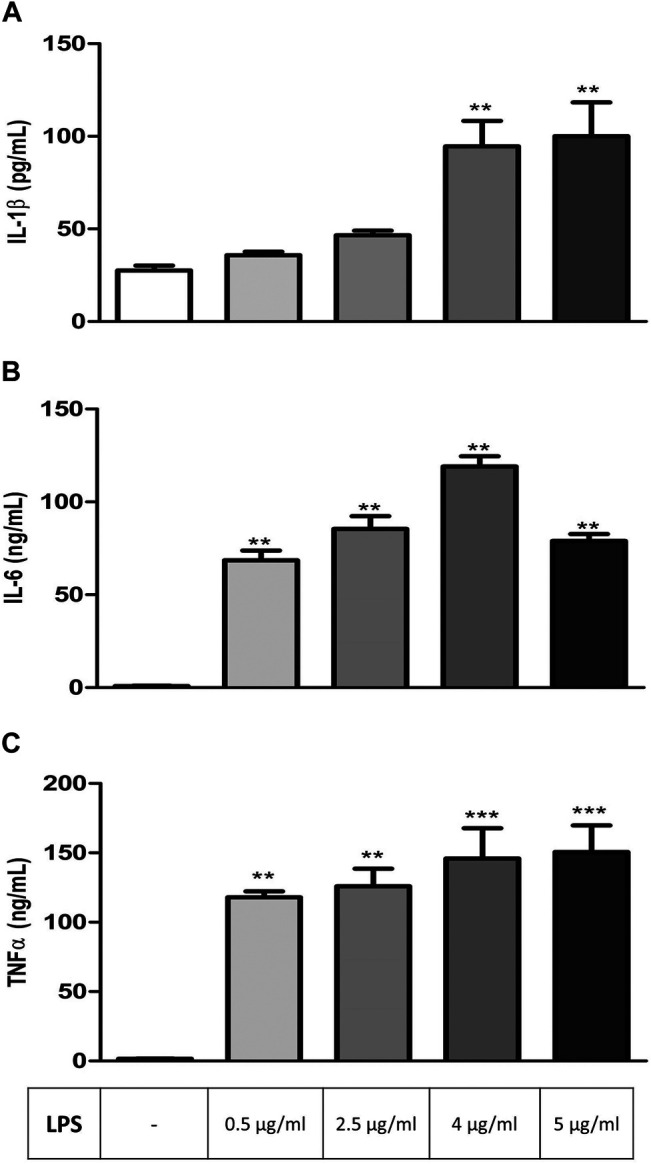
LPS-induced increase in inflammation markers in RAW264.7 cells. Shown are IL-1β **(A)**; IL-6 **(B)**, TNFα **(C)** levels in response to increasing LPS concentrations. Data are expressed as mean *±* S.E.M. (**p < 0.01; ***p < 0.001 vs. the control group, *n* = 5–7, One-way ANOVA with post hoc Tukey–Kramer multiple comparisons test or Student’s *t*-test).

RAW 264.7 cells were exposed to increasing concentrations of LPS (0.5, 2.5, 4, and 5 μg/ml) for 24 h before analyzing IL-1β, IL-6, and TNFα levels to determine the effective concentration of LPS to be used in other groups of experiments. All three parameters increased significantly depending on LPS concentration in comparison to controls, and the maximum effective concentration of LPS, which induces the secretion of all three cytokines, was found to be 4 μg/ml (Figures 1A–C).

**FIGURE 2:**
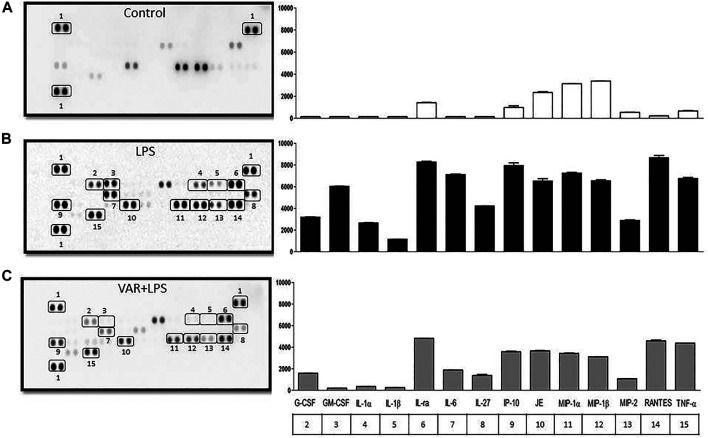
Effects of varenicline on LPS-induced cytokine levels in RAW 264.7 macrophages. Shown are membrane-based antibody arrays of 14 mouse cytokines found in supernatants of the control group **(A)**, 4 μg/ml LPS-induced **(B)** and LPS-induced macrophages in the presence of 1 µM varenicline (VAR) **(C)**. Each sample dot corresponds to a specific cytokine released from the same cell population used in other experiments (pooled data, *n* = 5–7). Bar graphics show averaged pixel intensities of each dot in duplicate. The table below the right panel shows the numerical annotations of the relevant cytokines detected in the left membranes (1: reference spot).

In the second set of experiments, RAW 264.7 cells were treated with varenicline (1 μΜ) prior to the incubation with LPS (4 μg/ml) for 24 h. Cytokine array was performed to monitor 40 different cytokines released from the same cell populations used in experimental protocols in order to find the target inflammatory cytokines whose levels were affected by LPS and varenicline administration. Cytokine array showed that LPS elevated at least 14 out of 40 cytokines, including macrophage inflammatory proteins (MIP-1α, the MIP-1β, and MIP-2), IL-1, IL-6, IL-27, TNFα, RANTES (regulated upon activation and normal T-cell expressed and secreted, also known as chemokine C-C motif ligand 5, CCL5), interferon gamma-induced protein 10 (IP-10), and monocyte chemoattractant protein (MCP-1 or JE) compared to the control group (Figures 2A,B). Varenicline decreased LPS-induced levels of these cytokines compared to the LPS group (each dot is in duplicate and represents 5–7 samples, pooled data, Figures 2A–C).

### Inhibitory Effects of Varenicline on LPS-Induced IL-1β, IL-6, and TNFα Elevations Determined by ELISA

**FIGURE 3:**
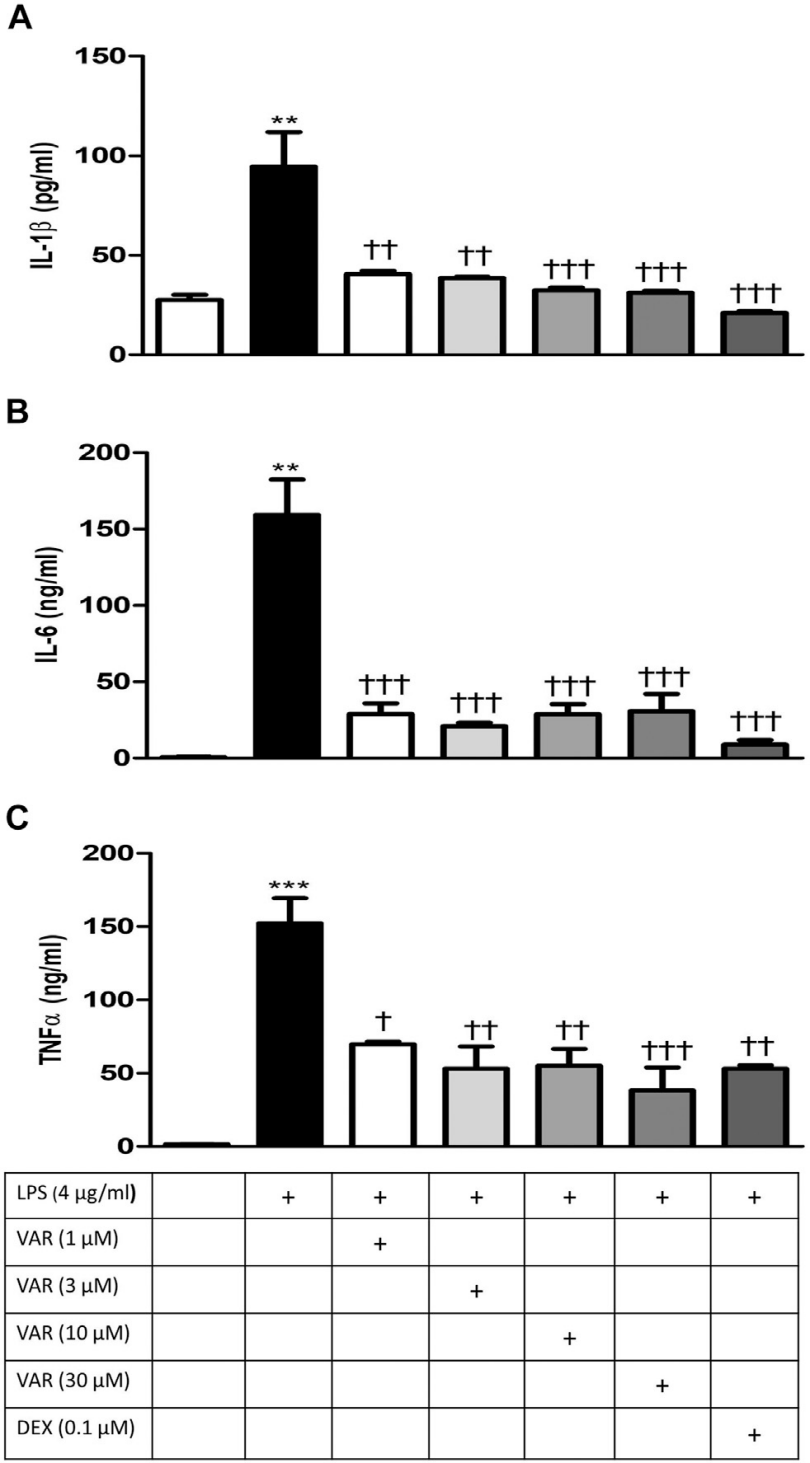
Effects of varenicline on LPS-induced IL-1β, IL-6, and TNFα elevations via nAChR. Shown are the effects of varenicline on 4 μg/ml LPS-induced IL-1β **(A)**, IL-6 **(B)**, and TNFα **(C)** levels and the comparison with dexamethasone. Data are expressed as mean ± S.E.M. (**, *p* < 0.01; ***, *p* < 0.001 vs. the control group; ^†^ *p <* 0.05; ^††^ *p* < 0.01, ^†††^ *p <* 0.001 vs. the LPS group, *n* = 5–7, One-way ANOVA with post hoc Tukey–Kramer multiple comparisons test or Student’s *t*-test). VAR: varenicline, DEX: dexamethasone.

RAW 264.7 cells were pretreated with increasing concentrations of varenicline (1-3-10-30 μΜ) 24 h prior to the administration of predetermined LPS concentration (4 μg/ml). Varenicline suppressed LPS-elevated IL-1β, IL-6, and TNFα levels (Figures 3A–C). Higher concentrations of varenicline (>1 µM) did not inhibit IL-6 and TNFα levels further. LPS-induced IL-1β, IL-6, and TNFα elevations were also suppressed by dexamethasone to a similar extent as observed with varenicline (Figures 3A–C). Levels of these three parameters were not altered by varenicline or dexamethasone treatment *per se* (not shown).

### nAChR-Mediated Suppression of LPS-Induced IL-1β, IL-,6 and TNFα Levels by Varenicline

**FIGURE 4:**
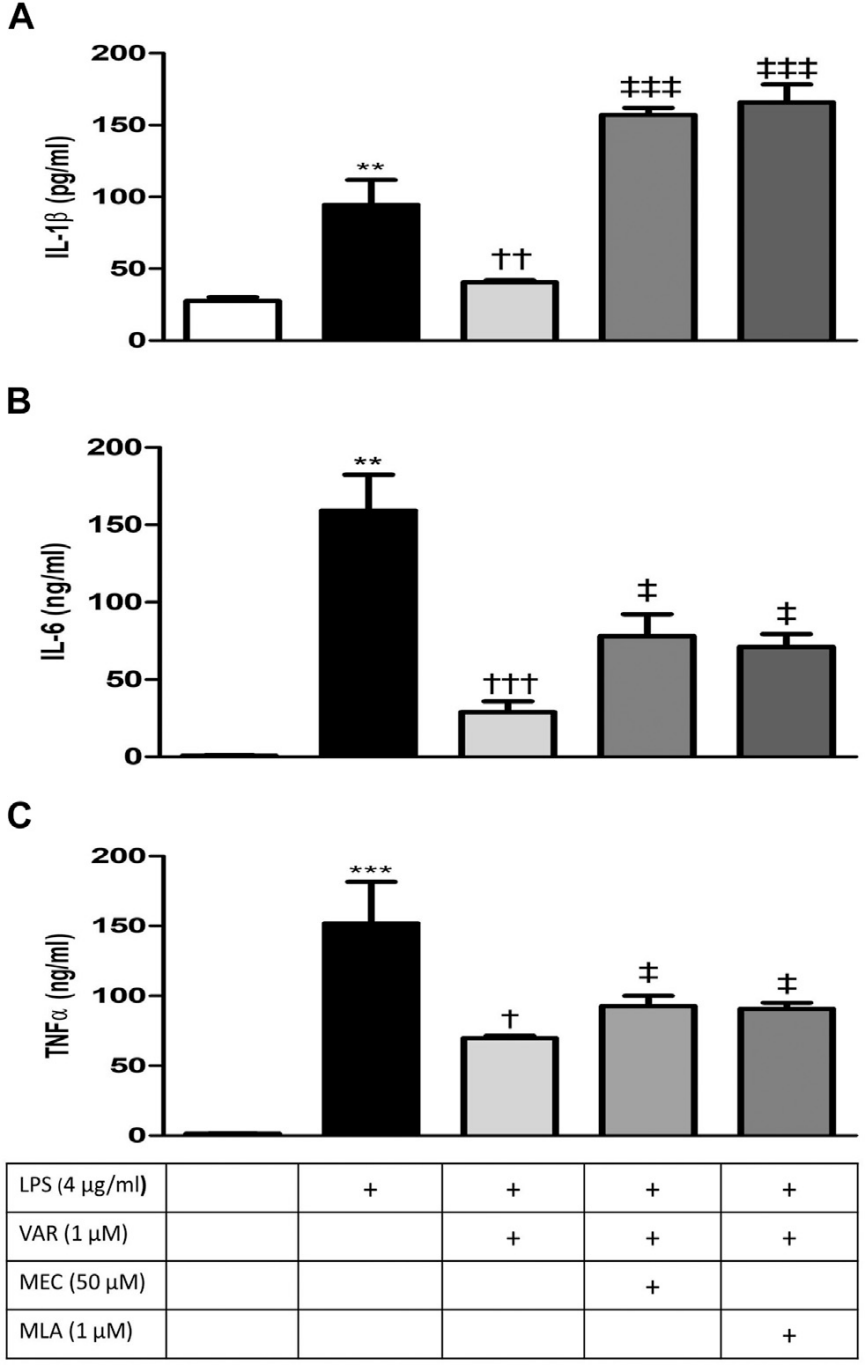
Effects of varenicline on LPS-induced IL-1β, IL-6, and TNFα elevations in the presence or absence of nAChR antagonists. Shown are 4 μg/ml LPS-elevated IL-1β **(A)**; IL-6 **(B)**, and TNFα **(C)** levels in the absence or presence of varenicline (VAR, 1 μM), mecamylamine (MEC, 50 μM) and methyllycaconitine (MLA, 1 μM). Data are shown as mean ± S.E.M. (**p < 0.01; ***p < 0.001 vs. the control group, ^†^ *p <* 0.05; ^††^ *p* < 0.01, ^†††^ *p <* 0.001 vs. LPS group; ^‡^ *p* < 0.05, ^‡‡‡^ *p* < 0.001 vs. LPS + VAR, *n* = 5–7, One-way ANOVA with post hoc Tukey–Kramer multiple comparisons test or Student’s t-test).

RAW 264.7 cells were pretreated with mecamylamine (MEC) and/or methyllycaconitine citrate (MLA) prior to incubation with varenicline (1 μΜ) and LPS (4 μg/ml) for 24 h. IL-1β, IL-6, and TNFα levels significantly increased in MEC and MLA groups compared to varenicline-treated groups (Figures 4A–C **)**. MEC or MLA did not alter IL-1β, IL-6, and TNFα levels *per se* (not shown).

### Inhibitory Effects of Varenicline on LPS-Induced Cell Proliferation

The cells were seeded and incubated in a biosafety cabinet for 30 min before the real-time proliferation assay. After 24 h of proliferation, the cells were treated with LPS (4 μg/ml) with or without varenicline (1 µM) in the first set of experiments. In the second set, the cells were pretreated with MEC and MLA prior to varenicline (1 μM) and LPS (4 μg/ml) administration. After drug treatment, changes in the cell proliferation rate (expressed as cell index, CI) were monitored for 24 h with 15-min intervals.

**FIGURE 5:**
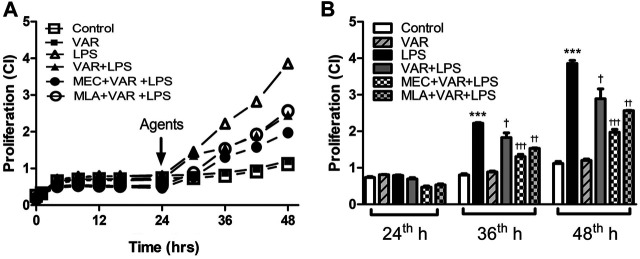
Effects of varenicline on LPS-induced cell proliferation in the presence or absence of nAChR antagonists. Shown are line graphs drawn from the averaged data points of the real-time proliferation assay tracings **(A)** and the cumulative data **(B)**. RAW 264.7 cells were treated with varenicline (VAR, 1 μM) in the presence or absence of mecamylamine (MEC, 50 μM) and methyllycaconitine (MLA, 1 μM) prior to lipopolysaccharide (LPS, 4 μg/ml) administration at 24th hour. Then, cell proliferation rates were monitored for 24 h after the treatments. Data are expressed as mean ± S.E.M. (***p < 0.01 vs. the control group ^†^ *p <* 0.05; ^††^ *p* < 0.01, ^†††^ *p <* 0.001 vs. the LPS group, *n* = 5–7, One-way ANOVA with post hoc Tukey–Kramer multiple comparisons test or Student’s *t*-test). CI: cell index.

The cell proliferation rate at 36^th^ and 48^th^ hours significantly increased in LPS groups (Figures 5A,B). The proliferation rate at 36^th^ and 48^th^ hours was not altered by varenicline significantly, indicating that varenicline has no cytotoxic effects at given concentrations (Figure 5A). Varenicline significantly decreased the LPS-induced cell proliferation rate at 36^th^ and 48^th^ hours (Figure 5B). However, MEC and MLA treatment did not antagonize the inhibitory effects of varenicline on LPS-induced proliferation (Figure 5B). MEC or MLA administration had no effect on the basal proliferation rate *per se* (not shown).

### Inhibitory Effects of Varenicline on LPS-Induced Cell Migration

The cells were seeded for 30 min prior to the experiment and then pretreated with MEC and MLA prior to varenicline (1 μΜ) and LPS (4 μg/ml) administration. After drug treatment, the change in the cell migration rate (expressed as cell index, CI) was monitored for 24 h at 15-min intervals.

**FIGURE 6:**
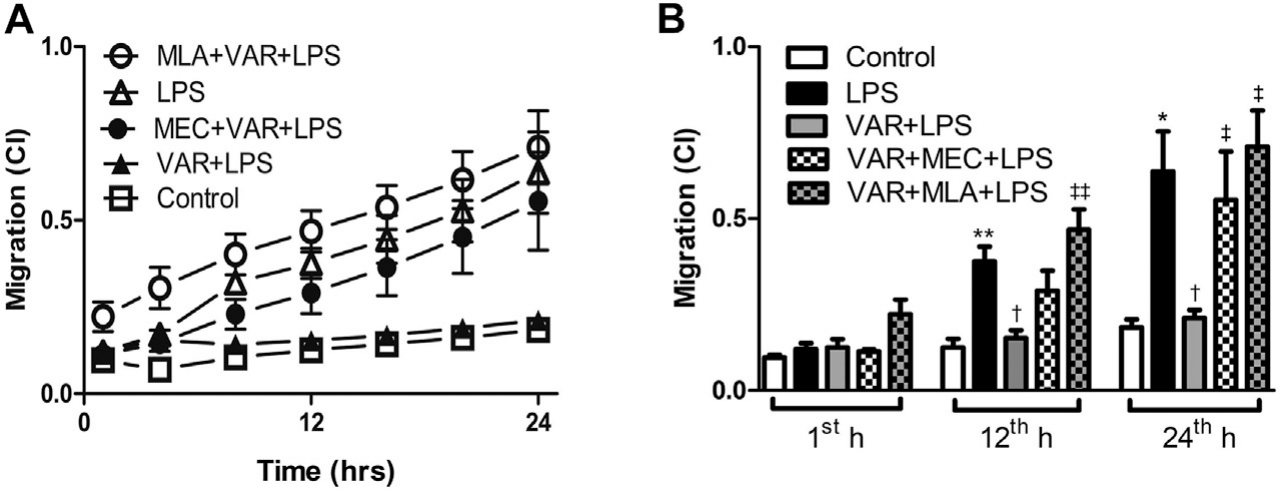
Effects of varenicline on LPS-induced cell migration in the presence or absence of nAChR antagonists. Shown are line graphs drawn by the averaged data points of real-time migration assay tracings **(A)** and the cumulative data **(B)**. RAW 264.7 cells were treated with varenicline (VAR, 1 μM) in the presence or absence of mecamylamine (MEC, 50 μM) and methyllycaconitine (MLA, 1 μM) prior to lipopolysaccharide (LPS, 4 μg/ml) administration at the 24th hour. Then, cell migration rates were monitored for 24 h after the treatments. Data are expressed as mean *±* S.E.M. (*p < 0.05; **p < 0.01 vs. the control group; ^†^ *p <* 0.05 vs. the LPS group; ^‡^ *p* < 0.05, ^‡‡^ *p <* 0.01 vs. LPS + VAR group, *n* = 5–7, One-way ANOVA with post hoc Tukey–Kramer multiple comparisons test or Student’s *t*-test).

The cell migration rate significantly increased in LPS groups compared to control group at 12^th^ and 24^th^ hours (*p* < 0.01, Figure 6). Varenicline decreased LPS-induced cell migration comparable to LPS groups at 12^th^ and 24^th^ hours (*p* < 0.01, Figure 6). Either MLA or MEC abolished varenicline’s inhibitory effects on LPS-induced cell migration compared to varenicline-treated groups at 12^th^ and 24^th^ hours (*p* < 0.01, Figure 6).

## Discussion

This study shows that varenicline significantly inhibits LPS-induced pro-inflammatory cytokine levels via α7nAChR along with concomitant cell proliferation and migration, suggesting its potential value in the prevention of inflammation by activating the cholinergic anti-inflammatory pathway. Currently used as a safe treatment option as a smoking cessation aid, varenicline has full agonistic properties on α7nAChRs (Mihalak et al., 2006a; Hays et al., 2008). A limited number of studies suggested a possible anti-inflammatory effectiveness of varenicline (Chen et al., 2017; Koga et al., 2018; McElroy et al., 2018). LPS is known to trigger inflammation by inducing the production of cytokines, including tumor necrosis factor (TNFα) and interleukins (Pavlov et al., 2003; Parrish et al., 2008; Yi et al., 2015). While moderately limiting local infections at the beginning, cytokines at higher levels lead to a widespread anti-inflammatory response (i.e., cytokine storm). In this regard, the intracellular endosomes of mononuclear cells might need a secondary induction by ATP to release IL-1β further after LPS stimulation (Turola et al., 2012; Stoffels et al., 2015). Therefore, in the first part of this study, RAW 264.7 macrophages were treated with increasing concentrations of LPS to determine its optimal concentration (4 μg/ml) for inducing cytokine release, including IL-1β.

Varenicline has also been shown to decrease inflammation in lung tissues of animals with emphysema (Koga et al., 2018) and to reduce brain inflammation in animals with stroke (Chen et al., 2017). A clinical study has also demonstrated that varenicline significantly decreased eicosanoid-related inflammation and oxidative damage in patients during smoking cessation therapy (McElroy et al., 2018). Consistent with these, our study showed that varenicline decreased LPS-induced inflammatory cytokine levels in RAW 264.7 macrophage cells. It is known that glucocorticoids are potent anti-inflammatory agents that modulate inflammation response through the attenuation of the cytokine release. Dexamethasone has been shown to decrease LPS-induced cytokine release in RAW 264.7 cells (Jeon et al., 2000; Ai et al., 2020). Our results showed no significant differences between varenicline and dexamethasone regarding the inhibition of IL-1β, IL-6, and TNFα levels. In brief, regardless of the mechanism of action, the extension of varenicline’s anti-inflammatory properties is similar to that of dexamethasone; however, its efficacy should also be confirmed *in vivo.*

Nicotinic receptors play important roles in the development of pain and inflammation associated with inflammatory pain models (Bagdas et al., 2015). The increase in cytokine levels in the presence of nAChR antagonists, MEC, and MLA, suggests the α7nAChR involvement in the anti-inflammatory effects of varenicline. Varenicline’s anti-inflammatory effectiveness in lung and brain tissues in mice with emphysema and stroke has been shown to occur by α7nAChR activation (Chen et al., 2017; Koga et al., 2018). These studies provide indirect evidence for the anti-inflammatory role of varenicline without investigating its effects on inflammatory cytokine levels. Accumulating evidence suggests that α7nAChRs expressed on immune cells are required to balance the endogenous response to inflammation through the activation of the cholinergic system (Pavlov et al., 2003). Agents acting on α7nAChRs have been shown to inhibit LPS-induced inflammatory response in various *in vivo* and *in vitro* studies (Pavlov et al., 2003; Wang et al., 2003; Yilmaz et al., 2006; Pavlov et al., 2007; Parrish et al., 2008; Yi et al., 2015; Koga et al., 2018). Several molecular mechanisms have been suggested for the α7nAChR-mediated inhibition of pro-inflammatory cytokines in macrophages, such as inhibiting the nuclear translocation of the transcription factor NF-κB and JAK2/STAT3 signaling pathway. As found among ligand-gated Ca^2+^ channels, the activation of a7nAChRs leads to the phosphorylation of AKT by JAK2 and PI3K upon calcium influx (Tsurutani et al., 2005; Chatterjee et al., 2009; Marrero and Bencherif, 2009) Our data provide experimental evidence that the α7nAChR agonist varenicline suppresses inflammatory cytokine release through a receptor-dependent mechanism and may have a therapeutic potential as an agent acting on the cholinergic anti-inflammatory pathway, although its downstream intracellular mechanisms were not investigated in the present study. Anti-inflammatory effects of varenicline may also be potentiated by decreases in LPS-elevated macrophage inflammatory proteins (MIP-1α, the MIP-1β, and MIP-2) (present study) which cause IL-1, IL-6, and TNFα release from macrophages besides their chemotactic properties (Bird and Kirstein, 2009; Selli et al., 2016).

The basal proliferation rate unaltered by varenicline may also indicate that the drug is not cytotoxic at effective concentrations. Additionally, varenicline treatment decreased LPS-induced cell proliferation, without any nAChR involvement. Studies suggest that the α7nAChR expression was associated with an increase in cell proliferation in breast, gastric, and lung cancer epithelial cells (Dang et al., 2016). Another study showed that α7nAChR agonist GTS-21 only partially decreased LPS-induced macrophage cell proliferation at early LPS response (Khan et al., 2012). These evidences suggest that nicotinic receptors may only play a partial role in LPS-induced macrophage proliferation. During inflammation, macrophage proliferation and differentiation are mediated by the LPS-elevated levels of granulocyte colony-stimulating factor (G-CSF) and granulocyte macrophage CSF (GM-CSF) (Li et al., 2014; Lee et al., 2017). Cytokine array showed that the suppression of LPS-induced G-CSF and GM-CSF levels by varenicline treatment might be involved in slowing the proliferation rate of RAW 264.7 cells (the present study).

LPS-induced TNFα, IL-1β, and IL-6 facilitate macrophage migration (Bozza et al., 1999; Toh et al., 2006; Yang et al., 2015). Acetylcholine has been shown to inhibit LPS-induced matrix metalloproteinase 9 (MMP-9) production and macrophage migration through α7nAChR activation (Yang et al., 2015). Similarly, α7nAChRs were found to be operational in LPS-induced macrophage migration, along with decrease in cytokine levels at non-cytotoxic varenicline concentrations. Our cytokine array data suggest that varenicline’s antimigratory effects may also depend on the decreased levels of RANTES, macrophage inflammatory proteins (MIP-1α, and MIP-1β and MIP-2), interferon–gamma inducible protein of 10 kDa (IP-10), and monocyte chemoattractant protein (MCP-1 or JE) (Lee et al., 1990; Nakata et al., 1991; Byrnes et al., 1999; Arima et al., 2000; Tajima et al., 2008). In addition to their chemotactic properties, MIPs have pro-inflammatory effects that induce IL-1, IL-6, and TNFα release from macrophages (Mühl and Dinarello, 1997; Haberstroh et al., 2002; Tsurutani et al., 2005; Deshmane et al., 2009; Marrero and Bencherif, 2009; Bandow et al., 2012), also confirmed by the present study as varenicline treatment decreased MIP-1α, MIP-1β, and MIP-2 levels, along with other cytokines.

## Limitations

Investigating the intracellular mechanism of varenicline on inflammatory response was beyond the scope of the present study. Although apparently relevant at the pre-clinical level, *in vivo* studies should be performed to confirm varenicline’s inhibitory effects on LPS-induced cytokine release via α7nAChR activation*.*

## Conclusion

Inflammation is a complicated process that requires specific and diverse treatment strategies. This study originally demonstrates in a model of LPS-induced inflammation using murine macrophage cell lines that varenicline stimulates the cholinergic anti-inflammatory pathway through α7nAChRs, resulting in the inhibition of cell migration and reduction of inflammatory cytokine secretion. Clarification of the underlying mechanism of action in different experimental models including the inflammatory state may contribute to the validation of varenicline’s efficacy for repurposing as an anti-inflammatory agent.

## Data Availability

The raw data supporting the conclusions of this article will be made available by the authors, without undue reservation.

## References

[B1] Ai F. Zhao G. Lv W. Liu B. Lin J. (2020). Dexamethasone Induces Aberrant Macrophage Immune Function and Apoptosis. Oncol. Rep. 43, 427–436. 10.3892/or.2019.7434 31894280 PMC6967116

[B2] Arima K. Nasu K. Narahara H. Fujisawa K. Matsui N. Miyakawa I. (2000). Effects of Lipopolysaccharide and Cytokines on Production of RANTES by Cultured Human Endometrial Stromal Cells. Mol. Hum. Reprod. 6, 246–251. 10.1093/molehr/6.3.246 10694272

[B3] Bagdas D. AlSharari S. D. Freitas K. Tracy M. Damaj M. I. (2015). The Role of Alpha5 Nicotinic Acetylcholine Receptors in Mouse Models of Chronic Inflammatory and Neuropathic Pain. Biochem. Pharmacol. 97, 590–600. 10.1016/j.bcp.2015.04.013 25931144 PMC4600420

[B4] Bandow K. Kusuyama J. Shamoto M. Kakimoto K. Ohnishi T. Matsuguchi T. (2012). LPS-induced Chemokine Expression in Both MyD88-dependent and -independent Manners Is Regulated by Cot/Tpl2-ERK axis in Macrophages. FEBS Lett. 586, 1540–1546. 10.1016/j.febslet.2012.04.018 22673523

[B5] Bernik T. R. Friedman S. G. Ochani M. DiRaimo R. Ulloa L. Yang H. (2002). Pharmacological Stimulation of the Cholinergic Antiinflammatory Pathway. J. Exp. Med. 195, 781–788. 10.1084/jem.20011714 11901203 PMC2193742

[B6] Bird C. Kirstein S. (2009). Real-time, Label-free Monitoring of Cellular Invasion and Migration with the xCELLigence System. Nat. Methods 6, v–vi. 10.1038/nmeth.f.263

[B7] Borovikova L. V. Ivanova S. Zhang M. Yang H. Botchkina G. I. Watkins L. R. (2000). Vagus Nerve Stimulation Attenuates the Systemic Inflammatory Response to Endotoxin. Nature 405, 458–462. 10.1038/35013070 10839541

[B8] Bozza M. Satoskar A. R. Lin G. Lu B. Humbles A. A. Gerard C. (1999). Targeted Disruption of Migration Inhibitory Factor Gene Reveals its Critical Role in Sepsis. J. Exp. Med. 189, 341–346. 10.1084/jem.189.2.341 9892616 PMC2192995

[B9] Byrnes H. D. Kaminski H. Mirza A. Deno G. Lundell D. Fine J. S. (1999). Macrophage Inflammatory Protein-3 Beta Enhances IL-10 Production by Activated Human Peripheral Blood Monocytes and T Cells. J. Immunol. 163, 4715–4720. 10528169

[B10] Cano P. M. Vargas A. Lavoie J.-P. (2016). A Real-Time Assay for Neutrophil Chemotaxis. Biotechniques 60, 245–251. 10.2144/000114416 27177817

[B11] Chatterjee P. K. Al-Abed Y. Sherry B. Metz C. N. (2009). Cholinergic Agonists Regulate JAK2/STAT3 Signaling to Suppress Endothelial Cell Activation. Am. J. Physiology-Cell Physiol. 297, C1294–C1306. 10.1152/ajpcell.00160.2009 PMC2777398 19741199

[B12] Chen S. Bennet L. McGregor A. L. (2017). Delayed Varenicline Administration Reduces Inflammation and Improves Forelimb Use Following Experimental Stroke. J. Stroke Cerebrovasc. Dis. 26, 2778–2787. 10.1016/j.jstrokecerebrovasdis.2017.06.051 28797614

[B13] Dang N. Meng X. Song H. (2016). Nicotinic Acetylcholine Receptors and Cancer. Biomed. Rep. 4, 515–518. 10.3892/br.2016.625 27123240 PMC4840641

[B14] De Jonge W. J. Ulloa L. (2007). The Alpha7 Nicotinic Acetylcholine Receptor as a Pharmacological Target for Inflammation. Br. J. Pharmacol. 151, 915–929. 10.1038/sj.bjp.0707264 17502850 PMC2042938

[B15] Deshmane S. L. Kremlev S. Amini S. Sawaya B. E. (2009). Monocyte Chemoattractant Protein-1 (MCP-1): An Overview. J. Interferon Cytokine Res. 29, 313–326. 10.1089/jir.2008.0027 19441883 PMC2755091

[B16] Fujii T. Mashimo M. Moriwaki Y. Misawa H. Ono S. Horiguchi K. (2017). Expression and Function of the Cholinergic System in Immune Cells. Front. Immunol. 8, 1085. 10.3389/fimmu.2017.01085 28932225 PMC5592202

[B17] Haberstroh U. Pocock J. Gómez-Guerrero C. Helmchen U. Hamann A. Gutierrez-Ramos J. C. (2002). Expression of the Chemokines MCP-1/CCL2 and RANTES/CCL5 Is Differentially Regulated by Infiltrating Inflammatory Cells. Kidney Int. 62, 1264–1276. 10.1111/j.1523-1755.2002.kid572.x 12234296

[B18] Hays J. T. Ebbert J. O. Sood A. (2008). Efficacy and Safety of Varenicline for Smoking Cessation. Am. J. Med. 121, S32–S42. 10.1016/j.amjmed.2008.01.017 18342165

[B19] Jeon Y. J. Han S. H. Lee Y. W. Lee M. Yang K. H. Kim H. M. (2000). Dexamethasone Inhibits IL-1β Gene Expression in LPS-Stimulated RAW 264.7 Cells by Blocking NF-κB/Rel and AP-1 Activation. Immunopharmacology 48, 173–183. 10.1016/S0162-3109(00)00199-5 10936515

[B20] Khan M. A. S. Farkhondeh M. Crombie J. Jacobson L. Kaneki M. Martyn J. A. J. (2012). Lipopolysaccharide Upregulates α7 Acetylcholine Receptors. Shock 38, 213–219. 10.1097/SHK.0b013e31825d628c 22683726 PMC3399057

[B21] Koga M. Kanaoka Y. Tashiro T. Hashidume N. Kataoka Y. Yamauchi A. (2018). Varenicline Is a Smoking Cessation Drug that Blocks Alveolar Expansion in Mice Intratracheally Administrated Porcine Pancreatic Elastase. J. Pharmacol. Sci. 137, 224–229. 10.1016/j.jphs.2018.06.007 30042025

[B22] Lee J.-H. Kim B. Jin W. J. Kim H.-H. Ha H. Lee Z. H. (2017). Pathogenic Roles of CXCL10 Signaling through CXCR3 and TLR4 in Macrophages and T Cells: Relevance for Arthritis. Arthritis Res. Ther. 19, 163. 10.1186/s13075-017-1353-6 28724396 PMC5518115

[B23] Lee M.-T. Kaushansky K. Ralph P. Ladner M. B. (1990). Differential Expression of M-CSF, G-CSF, and GM-CSF by Human Monocytes. J. Leukoc. Biol. 47, 275–282. 10.1002/jlb.47.3.275 1689760

[B24] Li W. Yang S. Kim S. O. Reid G. Challis J. R. G. Bocking A. D. (2014). Lipopolysaccharide-induced Profiles of Cytokine, Chemokine, and Growth Factors Produced by Human Decidual Cells Are Altered by Lactobacillus Rhamnosus Gr-1 Supernatant. Reprod. Sci. 21, 939–947. 10.1177/1933719113519171 24429676 PMC4107568

[B25] Marrero M. B. Bencherif M. (2009). Convergence of Alpha 7 Nicotinic Acetylcholine Receptor-Activated Pathways for Anti-apoptosis and Anti-inflammation: Central Role for JAK2 Activation of STAT3 and NF-Κb. Brain Res. 1256, 1–7. 10.1016/j.brainres.2008.11.053 19063868

[B26] McElroy J. P. Carmella S. G. Heskin A. K. Tang M. K. Murphy S. E. Reisinger S. A. (2018). Effects of Cessation of Cigarette Smoking on Eicosanoid Biomarkers of Inflammation and Oxidative Damage. PLoS One 14, e0218386–12. 10.1371/journal.pone.0218386 PMC6599218 31251764

[B27] Mihalak K. B. Carroll F. I. Luetje C. W. Mihalak K. B. Carroll F. I. Luetje W. (2006). Varenicline Is a Partial Agonist at α4β2 and a Full Agonist at α7 Neuronal Nicotinic Receptors. Mol. Pharmacol. 70 (3), 801–805. 10.1124/mol.106.025130.therapies 16766716

[B28] Mihalak K. B. Carroll F. I. Luetje C. W. (2006). Varenicline Is a Partial Agonist at α4β2 and a Full Agonist at α7 Neuronal Nicotinic Receptors. Mol. Pharmacol. 70, 801–805. 10.1124/mol.106.025130 16766716

[B29] Mühl H. Dinarello C. A. (1997). Macrophage Inflammatory Protein-1 Alpha Production in Lipopolysaccharide-Stimulated Human Adherent Blood Mononuclear Cells Is Inhibited by the Nitric Oxide Synthase Inhibitor N(G)-monomethyl-L-arginine. J. Immunol. 159, 5063–5069. 9366434

[B30] Murdock J. L. Núñez G. (2016). TLR4: The Winding Road to the Discovery of the LPS Receptor. J.I. 197, 2561–2562. 10.4049/jimmunol.1601400 27638937

[B31] Nakata K. Akagawa K. S. Fukayama M. Hayashi Y. Kadokura M. Tokunaga T. (1991). Granulocyte-macrophage colony-stimulating Factor Promotes the Proliferation of Human Alveolar Macrophages *In Vitro* . J. Immunol. 147, 1266–1272. 1869822

[B32] Palsson-McDermott E. M. O’Neill L. A. J. (2004). Signal Transduction by the Lipopolysaccharide Receptor, Toll-like Receptor-4. Immunology 113, 153–162. 10.1111/j.1365-2567.2004.01976.x 15379975 PMC1782563

[B33] Parrish W. R. Rosas-Ballina M. Gallowitsch-Puerta M. Ochani M. Ochani K. Yang L.-H. (2008). Modulation of TNF Release by Choline Requires α7 Subunit Nicotinic Acetylcholine Receptor-Mediated Signaling. Mol. Med. 14, 567–574. 10.2119/2008-00079.Parrish 18584048 PMC2435495

[B34] Pavlov V. A. Ochani M. Yang L.-H. Gallowitsch-Puerta M. Ochani K. Lin X. (2007). Selective α7-nicotinic Acetylcholine Receptor Agonist GTS-21 Improves Survival in Murine Endotoxemia and Severe Sepsis*. Crit. Care Med. 35, 1139–1144. 10.1097/01.CCM.0000259381.56526.96 17334244

[B35] Pavlov V. A. Wang H. Czura C. J. Friedman S. G. Tracey K. J. (2003). The Cholinergic Anti-inflammatory Pathway: a Missing Link in Neuroimmunomodulation. Mol. Med. 9, 125–134. 10.1007/bf03402177 14571320 PMC1430829

[B36] Pinder N. Bruckner T. Lehmann M. Motsch J. Brenner T. Larmann J. (2019). Effect of Physostigmine on Recovery from Septic Shock Following Intra-abdominal Infection - Results from a Randomized, Double-Blind, Placebo-Controlled, Monocentric Pilot Trial (Anticholium Per Se). J. Crit. Care 52, 126–135. 10.1016/j.jcrc.2019.04.012 31035187

[B37] Pinder N. Zimmermann J. B. Gastine S. Würthwein G. Hempel G. Bruckner T. (2019). Continuous Infusion of Physostigmine in Patients with Perioperative Septic Shock: A Pharmacokinetic/pharmacodynamic Study with Population Pharmacokinetic Modeling. Biomed. Pharmacother. 118, 109318. 10.1016/j.biopha.2019.109318 31398669

[B39] Selli C. Erac Y. Tosun M. (2016). Effects of Cell Seeding Density on Real-Time Monitoring of Anti-proliferative Effects of Transient Gene Silencing. J. Biol. Res-thessaloniki 23, 1–8. 10.1186/s40709-016-0057-4 PMC5133759 27981039

[B40] Snider S. A. Margison K. D. Ghorbani P. LeBlond N. D. O'Dwyer C. Nunes J. R. C. (2018). Choline Transport Links Macrophage Phospholipid Metabolism and Inflammation. J. Biol. Chem. 293, 11600–11611. 10.1074/jbc.RA118.003180 29880645 PMC6065184

[B41] Stoffels M. Zaal R. Kok N. van der Meer J. W. M. Dinarello C. A. Simon A. (2015). ATP-induced IL-1Î² Specific Secretion: True under Stringent Conditions. Front. Immunol. 6, 54. 10.3389/fimmu.2015.00054 25729382 PMC4325933

[B42] Tajima T. Murata T. Aritake K. Urade Y. Hirai H. Nakamura M. (2008). Lipopolysaccharide Induces Macrophage Migration via Prostaglandin D2and Prostaglandin E2. J. Pharmacol. Exp. Ther. 326, 493–501. 10.1124/jpet.108.137992 18492946

[B43] Toh M.-L. Aeberli D. Lacey D. Yang Y. Santos L. L. Clarkson M. (2006). Regulation of IL-1 and TNF Receptor Expression and Function by Endogenous Macrophage Migration Inhibitory Factor. J. Immunol. 177, 4818–4825. 10.4049/jimmunol.177.7.4818 16982923

[B44] Tsurutani J. Castillo S. S. Brognard J. Granville C. A. Zhang C. Gills J. J. (2005). Tobacco Components Stimulate Akt-dependent Proliferation and NFκB-dependent Survival in Lung Cancer Cells. Carcinogenesis 26, 1182–1195. 10.1093/carcin/bgi072 15790591

[B45] Turola E. Furlan R. Bianco F. Matteoli M. Verderio C. (2012). Microglial Microvesicle Secretion and Intercellular Signaling. Front. Physio. 3, 149. 10.3389/fphys.2012.00149 PMC3357554 22661954

[B46] Wang H. Yu M. Ochani M. Amella C. A. Tanovic M. Susarla S. (2003). Nicotinic Acetylcholine Receptor α7 Subunit Is an Essential Regulator of Inflammation. Nature 421, 384–388. 10.1038/nature01339 12508119

[B47] Yang Y.-H. Li D.-L. Bi X.-Y. Sun L. Yu X.-J. Fang H.-L. (2015). Acetylcholine Inhibits LPS-Induced MMP-9 Production and Cell Migration via the A7 nAChR-Jak2/stat3 Pathway in RAW264.7 Cells. Cell. Physiol. Biochem. 36, 2025–2038. 10.1159/000430170 26202362

[B48] Yi L. Luo J.-f. Xie B.-b. Liu J.-x. Wang J.-y. Liu L. (2015). α7 Nicotinic Acetylcholine Receptor Is a Novel Mediator of Sinomenine Anti-inflammation Effect in Macrophages Stimulated by Lipopolysaccharide. Shock 44, 188–195. 10.1097/SHK.0000000000000389 25895149

[B49] Yilmaz Z. Ilcol Y. O. Torun S. Ulus I. H. (2006). Intravenous Administration of Choline or CDP-Choline Improves Platelet Count and Platelet Closure Times in Endotoxin-Treated Dogs. Shock 25, 73–79. 10.1097/01.shk.0000185796.04589.15 16369190

[B50] Zimmermann J. B. Pinder N. Bruckner T. Lehmann M. Motsch J. Brenner T. (2017). Adjunctive Use of Physostigmine Salicylate (Anticholium) in Perioperative Sepsis and Septic Shock: Study Protocol for a Randomized, Double-Blind, Placebo-Controlled, Monocentric Trial (Anticholium Per Se). Trials 18, 1–10. 10.1186/s13063-017-2231-x 29126416 PMC5681758

